# Type III IFN Receptor Expression and Functional Characterisation in the Pteropid Bat, *Pteropus alecto*

**DOI:** 10.1371/journal.pone.0025385

**Published:** 2011-09-27

**Authors:** Peng Zhou, Chris Cowled, Glenn A. Marsh, Zhengli Shi, Lin-Fa Wang, Michelle L. Baker

**Affiliations:** 1 Australian Animal Health Laboratory, CSIRO Livestock Industries, Geelong, Victoria, Australia; 2 State Key Laboratory of Virology, Wuhan Institute of Virology, Chinese Academy of Sciences, Wuhan, China; 3 Department of Microbiology and Immunology, The University of Melbourne, Parkville, Victoria, Australia; 4 Department of Biology, Center for Evolutionary and Theoretical Immunology, The University of New Mexico, Albuquerque, New Mexico, United States of America; National Institute on Aging, United States of America

## Abstract

Bats are rich reservoir hosts for a variety of viruses, many of which are capable of spillover to other susceptible mammals with lethal consequences. The ability of bats to remain asymptomatic to viral infection may be due to the rapid control of viral replication very early in the immune response through innate antiviral mechanisms. Type I and III interferons (IFNs) represent the first line of defence against viral infection in mammals, with both families of IFNs present in pteropid bats. To obtain further insight into the type III IFN system in bats, we describe the characterization of the type III IFN receptor (IFNλR) in the black flying fox, *P. alecto* with the characterization of IFNλR1 and IL10R2 genes that make up the type III IFN receptor complex. The bat IFNλR complex has a wide tissue distribution and at the cellular level, both epithelial and immune cells are responsive to IFN-λ treatment. Furthermore, we demonstrate that the bat IFNλR1 chain acts as a functional receptor. To our knowledge, this report represents the first description of an IFN receptor in any species of bat. The responsiveness of bat cells to IFN-λ support a role for the type III IFN system by epithelial and immune cells in bats.

## Introduction

The hallmark of the innate immune response to viral infection is the production of interferons (IFNs). IFNs play an essential role in the induction of an antiviral state and contribute to the initiation of the adaptive immune response. The type I IFNs (including IFN-α and IFN-β) are well known for their induction and potent antiviral activity directly in response to viral infection. More recently, a family of IFNs named type III IFNs (also known as IL28/29 or IFN-λs) were discovered in the human genome based on their similarity to type I IFN and IL-10 family members [1], [2]. Type I and III IFNs have similar antiviral activity, are produced by common pathways and result in the production of an overlapping repertoire of interferon stimulated genes (ISGs) [3]–[5]. Despite their similarities, type I and III IFNs signal through distinct IFN receptor (IFNR) complexes and display differences in their tissue and cellular distribution patterns consistent with each IFN family playing a distinct role in the immune response to viral infections.

Both type I and III IFNs induce their activity by signalling through a heterodimeric class II cytokine receptor. Type I IFNs signal through a receptor complex composed of IFNR alpha 1 (IFNAR1) and IFNAR2 chains while type III IFNs signal through a receptor composed of IFNλR1 (also known as IL-28Ra) and IL10R2 (also known as IL10Rb) chains [5]. The IFNλR1 chain serves as a unique subunit of the type III receptor complex and is critical to the specificity of ligand binding [5]. In contrast, IL10R2 also forms part of the receptor complex of several members of the IL-10 cytokine family, including IL-10, IL-22 and IL-26 [6]–[9]. The IL10R2 chain plays little role in ligand specificity but participates in the signalling cascade leading to the induction of ISGs.

The type III IFNR complex has only been characterized in humans and mice and in the amphibian, *Xenopus tropicalis* [1], [2], [10], [11]. Unlike the ubiquitously expressed type I IFNR complex, the type III IFNR has a more restricted tissue distribution pattern. Although the IL10R2 chain is ubiquitously expressed in all tissues and cells, the expression of the IFNλR1 varies widely between different organs and at the cellular level is restricted to epithelial cells [5], [12]–[14]. This expression pattern may reflect a role for type III IFNs in antiviral activity at the major portals of viral entry, providing a mechanism to inhibit viral replication prior to the activation of other components of the immune system. Although a high expression of IFNλR1 has also been detected in a variety of human and mouse immune cells, only mouse plasmacytoid dendritic cells have been shown to respond to IFN-λ [5], [12], [13], [15], [16].

Bats have been implicated as the natural reservoir for many of the most deadly viruses to have emerged in recent years, including SARS-Coronavirus (SARS-CoV), Ebola, Marburg, Hendra and Nipah virus. Bats generally show no symptoms of disease and appear to be capable of rapidly controlling viral replication to very low levels yet still allowing occasional spillover to other susceptible species [17]. Although the mechanisms responsible for the control of viral replication in bats are not understood, the co-evolution of bats with viruses may have resulted in the evolution of novel mechanisms for the control of viral replication. To elucidate the mechanisms responsible for the asymptomatic nature of viral infections in bats, we are using the black flying fox, *Pteropus alecto* as a model species for examining antiviral immunity in bats [18]–[21].

Previously we demonstrated that pteropid bats have two transcribed IFN-λ genes that are differentially induced relative to each other and to type I IFNs following dsRNA stimulation and viral infection. Bat IFN-λ2 also demonstrates significant antiviral activity *in vitro* [21]. As the biological activity of cytokines is mediated through binding to specific receptor complexes, information on cellular responses to cytokines provides important clues to biological function. Towards a better understanding of the bat type III IFN system, we report the molecular cloning and functional characterization of IFNλR1 and IL10R2 encoding the type III IFNR complex from *P. alecto*. Our results demonstrate that IFNλR1 has a wide tissue distribution with epithelial cells and immune cells responsive to IFN-λ in pteropid bats.

## Materials and Methods

### Cell culture

The generation of the *P. alecto* cell lines and culture conditions have been described previously [18], [21]. Six bat cell lines were used, including one cloned and immortalised kidney cell line (PaKiT01) and five primary cell lines originating from kidney, small intestine, brain, liver and lung, respectively [18]. Bat cell lines were cultured in DMEM/F12-Hams (Sigma), supplemented with 10% foetal calf serum (FCS, Hyclone), 100 units/ml penicillin, 100 mg/ml streptomycin and 50 mg/ml gentamycin (Sigma). All cells were maintained in a humidified atmosphere of 5% CO_2_ in air at 37°C.

The isolation of fresh splenocytes from *P. alecto* bats has been described previously [21]. Briefly, cell suspensions were prepared by pressing spleen tissue through a cell strainer using a syringe plunger. Mononuclear splenocytes were isolated by density centrifugation over lymphoprep (Axis-Shield). Culture media for mononuclear splenocytes consisted of DMEM supplemented with 10% FCS, 15 mM Hepes, 15 mM L-glutamine, 100 mg/ml penicillin and 100 mg/ml streptomycin. All animal experiments were approved by the Australian Animal Health Laboratory (AAHL) animal ethics committee (protocol number 1389).

### Cloning of IFN-λ receptor genes

Total RNA was extracted from *P. alecto* fresh spleen and thymus and polyI:C transfected PaLuT02 cells as described previously using the RNeasy mini kit (Qiagen) with on-column DNase-I treatment (Qiagen) [21]. Full-length coding sequences for IFNλR1 and IL10R2 were obtained using 5′ and 3′ rapid amplification of cDNA ends (RACE) polymerase chain reactions (PCRs). Reactions were performed on RNA extracted from polyI:C stimulated PaLuT02 cells using the GeneRacer Kit (Invitrogen, Carlsbad, CA, USA) with Long-amp DNA polymerase (New England Biosystems). Primers were designed based on *P. vampyrus* genome sequence information deposited in the Ensembl Genome Browser (assembly pteVam1, 2.633 coverage, July 2008) and are listed in Table S1. To obtain all potential isoforms of IFNλR1 and IL10R2, RT-PCR was also performed on RNA extracted from polyI:C stimulated PaLuT02 cells, spleen and thymus using primers listed in Table S1. To confirm the presence of introns within the 3′-UTR of IFN-λR1, PCR was performed on genomic DNA extracted from *P. alecto* liver using primers IFN-λR1-2F/6R listed in Table S1. The resultant RACE and PCR products were cloned and sequenced using the TOPO TA Cloning Kit (Invitrogen) and BigDye Terminator Cycle Sequencing Kit v3.1 respectively (Applied Biosystems, Foster City, CA, USA).

### Sequence analysis

Sequences were edited using SeqMan PRO (Lasergene) and assembled manually using Clone Manager 9.0 (Sci-Ed Software). To determine the intron-exon organization of *P. alecto* IFNλR1 and IL10R2 genes, the full length coding sequences were aligned with the corresponding sequences in the *P. vampyrus* genome. Intron-exon maps of the genes were drawn using Fancy Gene v1.4 (http://host13.bioinfo3.ifom-ieocampus.it/fancygene/). Putative protein sequences for bat IFNλR1 and IL10R2 were compared with sequences in the GenBank database using the BLASTP algorithm [22]. Sequence alignments were performed using the Clustal X program [23] and visualized using GeneDoc (www.nrbsc.org/gfx/genedoc/index.html). Transmembrane regions and Signal peptides were identified by TMHMM 2.0 and SignalP version 3.0 respectively (www.cbs.dtu.dk/services). The GenBank accession numbers for sequences used in the sequence alignment are as follows (amino acid/mRNA): NP_734464/NM_170743, *Homo sapiens* (human); NP_777276/NM_174851, *Mus musculus* (mouse); XP_001917953/XM_001917918, *Equus caballus* (horse).

### Quantitative reverse transcription PCR (qRT-PCR)

cDNA from ten fresh *P. alecto* tissues, including lymph nodes, PBMC, brain, heart, kidney, liver, spleen, small intestine, lung and salivary gland from three individual bats were prepared previously [20]. RNA from IFN-λ2 treated bat PaKiT02 cells and primary cells lines derived from kidney, small intestine, brain, liver and lung was also extracted as described for RACE PCR. qRT-PCR primers for IFNλR1 and IL10R2 were designed using Primer Express 3.0 (Applied Biosystems) with default parameter settings and are listed in Table S1. Primers for ISG56, RIG-I and 18s rRNA have been described previously [20], [21]. Reactions were carried out using EXPRESS SYBR® GreenER™ qPCR Supermix Universal (Invitrogen) for cDNA and SuperScript® III Platinum® SYBR® Green One-Step qRT-PCR Kit (Invitrogen) for RNA in an Applied Biosystems 7500 Fast Real-Time qPCR instrument. For each reaction from cDNA, 2 µl of 1∶5 diluted cDNA were used while in a reaction from RNA, 2 µl RNA per well were used. A final concentration of 200nmol each primer was used in these reactions.

The cycling profile for cDNA samples consisted of an initial denaturation at 90°C for 1 minute followed by 40 cycles of 90°C for 15 seconds, 60°C for 1 minute followed by melt curve analysis. For that of RNA, an initial reverse transcription step at 50°C for 2 min was added. Expression level of the target genes were calculated using either standard curves method (IFNλR1 and IL10R2) or fold induction compared to the mock (ISG56 and RIG-I). All data were normalised relative to the housekeeping gene 18s rRNA.

### Treatment of the bat cells with recombinant IFN-λ2

IFN-λ2 containing supernatant derived from a stable cell line expressing IFN-λ2 in 293T cells [21], was used to treat the bat cells. Bat cloned kidney cells, PaKiT01 and primary bat cell lines from kidney, small intestine, brain, liver, lung and fresh splenocytes were treated with 1∶2 diluted IFN-λ2 containing supernatant. This dilution was chosen based on the antiviral activity of recombinant IFN-λ2 described previously [21]. Supernatant from normal cultured 293T cells was used as mock control. Cells were incubated at 37°C for six hours and collected into buffer RLT (Qiagen) for extraction of total RNA.

### Plasmid construction and transfection

The bat IFNλR1 full-length open reading frame (ORF) was cloned into the pCAGGS transient expression vector with an in-frame C-terminal 6×His tag using the primers listed in Table S1. Correct cloning was confirmed by sequencing. The plasmid was prepared using PureYield kit (Promega) before transfection. Approximately 1×10^5^ bat PaKiT01 cells were seeded into 24-well plates. The plasmid was transfected into PaKiT01 cells using the Neon electroporation transfection system (Invitrogen). Before transfection, bat PaKiT01 cells were resuspended. All transfection reactions were performed according to the manufacturer's instructions.

## Results

### Characterization of IFN-λ receptor genes

IFNλR1 and IL10R2 were identified by key word searches of the low coverage whole genome sequence of the pteropid bat, *P. vampyrus* in the publicly available Ensembl database using the terms IL28Ra and IL10Rb. The regions containing the IL10R2 and IFNλ R1 genes in the current *P. vampyrus* whole genome assembly shared a high degree of conserved synteny with the corresponding regions in the human and mouse genomes. Previously identified immune genes from *P. alecto* and *P. vampyrus* including IFNs and TLRs have a high sequence similarity to each other [20], [21]. IFN-λ 2 genes for example, share 98% nucleotide identity between the two species. In view of the high sequence similarity between genes previously identified in *P. vampyrus* and *P. alecto* [20], [21], IFNλR sequences from *P. vampyrus* were used to design oligonucleotide primers to amplify full length cDNAs from *P. alecto.* RACE PCR was performed on RNA extracted from the bat lung cell line, PaLuT02, three hours following stimulation with polyI:C [21]. As soluble variants of IFNλR1 and IL10R2 have been identified in other species, RT-PCR was also performed on *P. alecto* spleen and thymus RNA, however, no additional isoforms were identified. Alignment of cDNA sequences with the *P. vampyrus* genome and genes from the other species available in the GenBank database, revealed the presence of a single form of IFNλR1 and IL10R2 in *P. alecto*. These sequences have been deposited into Genbank under accession numbers: JN000223 (IFNλR1) and JN000224 (IL10R2).

**Figure 1:**
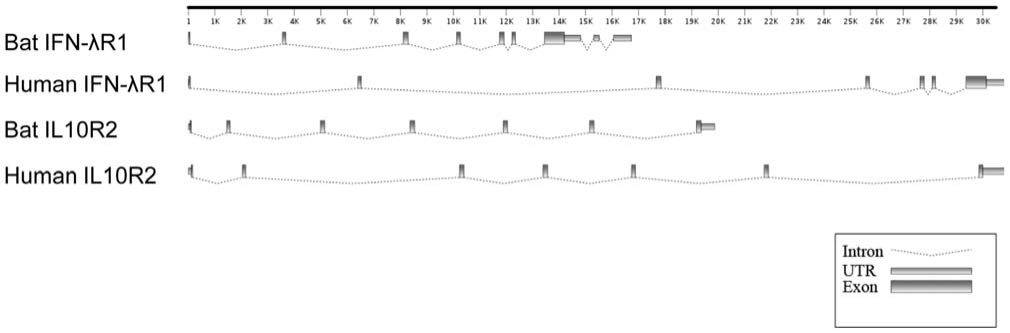
Gene organization of *P. alecto* IFNλR1 and IL10R2 compared with that of corresponding human genes. The intron-exon structures were predicted by nucleotide alignment of IFNλR1 or IL10R1 mRNA with the corresponding sequences in either the *P. vampyrus* or human genome sequences. Putative UTRs and exons are drawn as rectangles; introns are shown as dotted lines.

The genomic organization of each locus was determined by aligning the complete cDNA sequences of IFNλ1 and IL10R2 with the corresponding *P. vampyrus* genomic sequence (Fig. 1). Similar to human IFNλR1, the bat IFNλR1 locus contains seven exons and six introns in the region corresponding to the open reading frame (ORF). However, bat IFNλR1 is unusual in that it contains two additional introns in the 3′-UTR region. The presence of these introns in the 3′-UTR of IFNλR1 was further confirmed by PCR using *P. alecto* genomic DNA. The bat IL10R2 locus shares the same genomic structure as human IL10R2, with seven exons and six introns. As shown in Figure 1, the two bat IFNλR loci contain several introns that are shorter than those present in the corresponding human loci, resulting in the bat loci being more constrained in size. Human IFNλR1 and IL10R2 each span approximately 30kb compared with the bat loci which are 17kb and 20kb respectively (Fig. 1).

**Figure 2:**
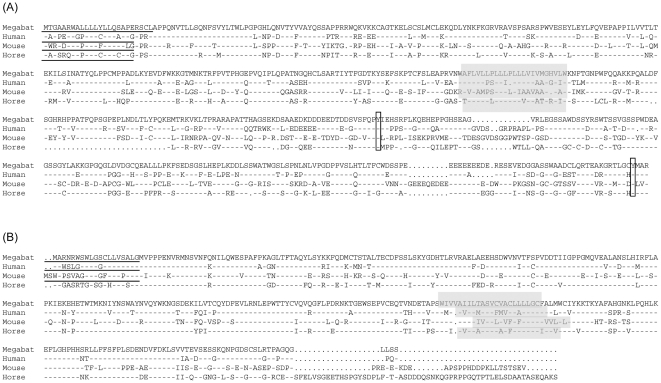
Alignment of the deduced amino acid sequence of *P. alecto* IFNλR1 (A) and IL10R2 (B) genes with human, mouse and horse IFNλR sequences. Signal peptides are underlined and transmembrane regions are shaded. The conserved cysteine and tyrosine residues are in bold. Two of the tyrosine residues in IFNλR1, which are involved in meditation of STAT2 tyrosine phosphorylation in human IFNλR1 are conserved in bat IFN-λR1 and are further boxed. *Dashes* indicated similarity and *dots* indicate gaps.

An alignment of the full length deduced protein sequences of bat IFNλR1 and IL10R2 with sequences from other species is shown in Figures 2A and 2B. Deduced proteins encoded by IFNλR1 and IL10R2 contained many of the conserved features present in human genes. Bat IFNλR1 contained an ORF of 1548 bp, encoding a protein of 515 amino acids (aa) with a 25 aa putative signal peptide (Fig. 2A). A 23 aa transmembrane region encoded by exon six separates the protein into a 203 aa extracellular domain and 265 aa intracellular domain. The deduced protein sequence was highly conserved with that of other mammals including the presence of six conserved cysteine residues in the extracellular region and three tyrosines in the intracellular region, two of which (Y344 and Y512) are essential for STAT2 and STAT5 activation [24]. The *P. alecto* IFNλR1 sequence shared 66–79% nucleotide identity and 50-65% amino acid identity with IFNλR1 genes from other mammals (Table 1).

**Table 1:** Identities of Megabat IFNλR1 with human, mouse and horse.

	Bat-IFNλR1	Human-IFNλR1	Mouse-IFNλR1	Horse-IFNλR1
Bat-IFNλR1		**69**	**54**	**65**
Human-IFNλR1	79		**57**	**63**
Mouse-IFNλR1	66	69		**50**
Horse-IFNλR1	75	74	64	

Bold numbers indicate amino acid identity; non-bold numbers represent nucleotide identity.

Bat IL10R2 contained an ORF of 984 bp, encoding a 327 aa protein. The 24 aa transmembrane region (221 to 244 aa) encoded by exon 6 separated the protein into a 201 aa extracellular region and 83 aa intracellular region. These features are highly conserved with human IL10R2. Bat IL10R2 has four conserved cysteine residues in the extracellular region and two conserved tyrosine residues in the intracellular region (Fig. 2B). The *P. alecto* IL10R2 sequence shared 75-85% nucleotide identity and 63–78% amino acid identity with IL10R2 genes from other mammals (Table 2).

**Table 2:** Identities of Megabat IL10R2 with human, mouse and horse.

	Bat-IL10R2	Human-IL10R2	Mouse-IL10R2	Horse-IL10R2
Bat-IL10R2		**82**	**69**	**78**
Human-IL10R2	85		**69**	**78**
Mouse-IL10R2	75	76		**63**
Horse-IL10R2	84	83	72	

Bold numbers indicate amino acid identity; non-bold numbers represent nucleotide identity.

### IFNλR1 and IL10R2 are transcribed in a variety of organs in *P. alecto*

**Figure 3:**
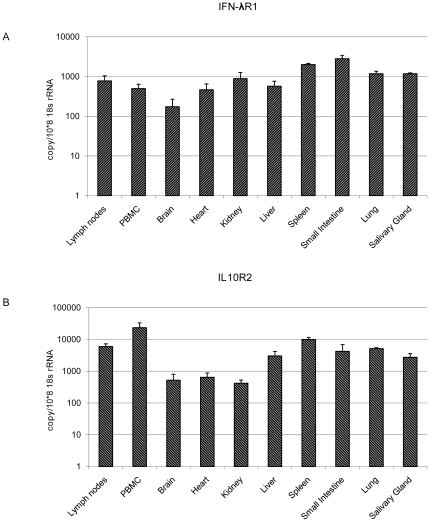
qRT-PCR detection of IFNλR1 (A) and IL10R2 (B) mRNA expression in *P. alecto*. PBMC: peripheral blood mononuclear cells. The expression level was normalized to housekeeping gene 18s rRNA. n = 3 individual apparently healthy wild-caught bats. The error bars represent standard deviation in (A) and (B).

To examine the tissue distribution of bat IFNλR1 and IL10R2, we determined their transcription in a range of *P. alecto* primary organs. As show in Figure 3A, IFNλR1 is transcribed in all organs tested with small intestine and spleen showing the highest transcription and brain the lowest. IL10R2 also appeared to be ubiquitously expressed, however, a higher level of variation between organs was detected in the expression of IL10R2 compared with IFNλR1 (Fig. 3B). The three immune related cells and organs, PBMC, spleen and lymph nodes had the highest expression of IL10R2. Brain, heart and kidney showed much lower transcription levels of IL10R2 compared to the other organs tested (Fig. 3B).

### IFN-λ targets epithelial cells in non- hematopoietic cell lines

**Figure 4:**
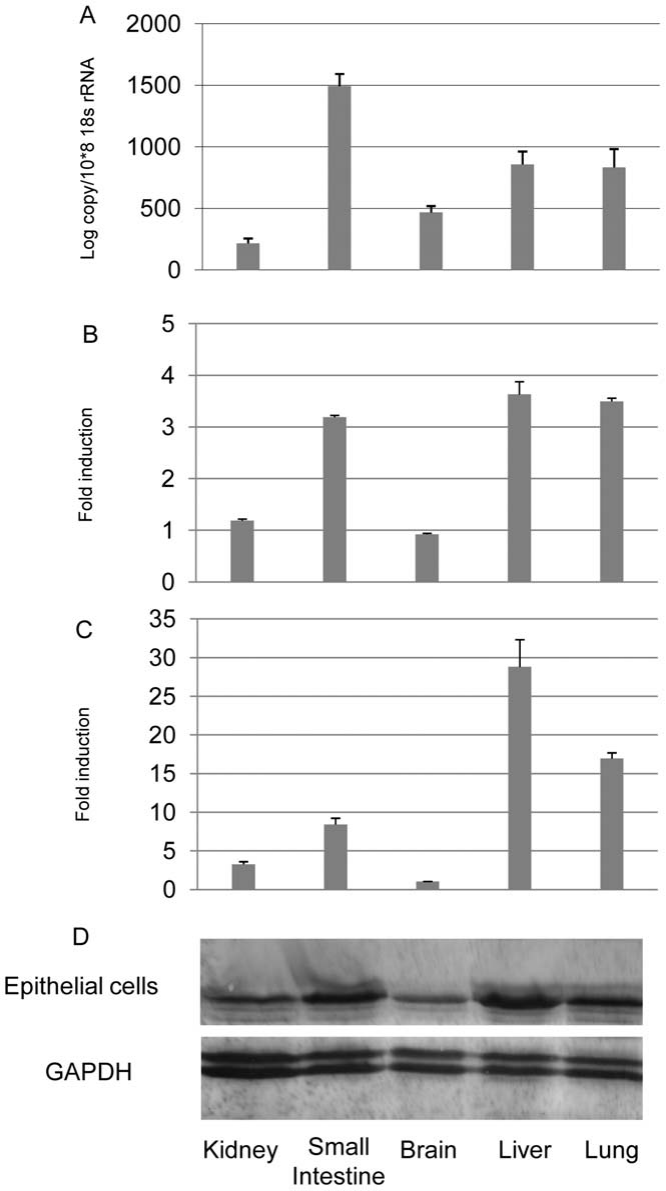
The responsiveness of *P. alecto* non-haematopoietic cell lines to IFN-λ2 treatment correlates with IFNλR1 expression and proportions of epithelial cells. (A) Relative expression level of IFNλR1 by qRT-PCR (mean of two experiments). The expression level was normalized to the housekeeping gene 18s rRNA. (B/C) Responsiveness of *P. alecto* kidney, small intestine, brain, liver and lung primary cells. The histogram shows the (B) RIG-I or (C) ISG56 fold induction in response to IFN-λ2 treatment (mean of two experiments). The error bars represent standard errors in (A), (B) and (C).(D) The relative proportion of epithelial cells in the primary cell cultures as demonstrated by Western blotting using an anti-cytokeratin antibody. The housekeeping gene, GAPDH was used as a loading control.

In view of the ubiquitous tissue distribution of bat IFNλR1, we next sought to determine the sensitivity of cell lines derived from different *P. alecto* tissues to IFN-λ treatment. In humans, the IFNλR1 chain is unique to the IFNλR complex and has substantially higher ligand affinity than the IL10R2 chain [3], [6], [7], [14]. We therefore focused on IFNλR1 to obtain information specific to the IFN-λ response. Bat primary cells, including five non-hematopoietic cell lines derived from kidney, small intestine, brain, liver and lung were examined for their response to recombinant bat IFN-λ2 [21]. As shown in Figure 4, there is a correlation between the expression of IFNλR1 (Fig. 4A) and the induction of the two ISGs tested, RIG-I (Fig. 4B) and ISG56 (Fig. 4C) following IFN-λ stimulation. Liver, lung and small intestine which have relatively higher IFNλR1 expression show correspondingly high ISGs induction. In contrast, kidney and brain responded poorly to IFN-λ, due to their low IFNλR1 expression.

The bat primary cell lines consist of a mixed population of adherent tissue cell types and are not a direct reflection of the original tissue they were derived from. In humans, the IFNλR1 expression among non-hematopoietic cells is restricted to epithelial cells [5]. To determine whether a similar pattern exists in bats we used a cross-specific epithelial cell marker (anti-cytokeratin) to determine the proportion of epithelial cells in each cell line population. The Western blot shown in Figure 4D illustrates the relative proportion of epithelial cells in each cell line population and demonstrates a correlation between the proportion of epithelial cells within the cell line populations, their IFNλR1 expression and ISGs production (Fig. 4).

### Immune cells express IFNλR1 and respond to IFN-λ treatment

**Figure 5:**
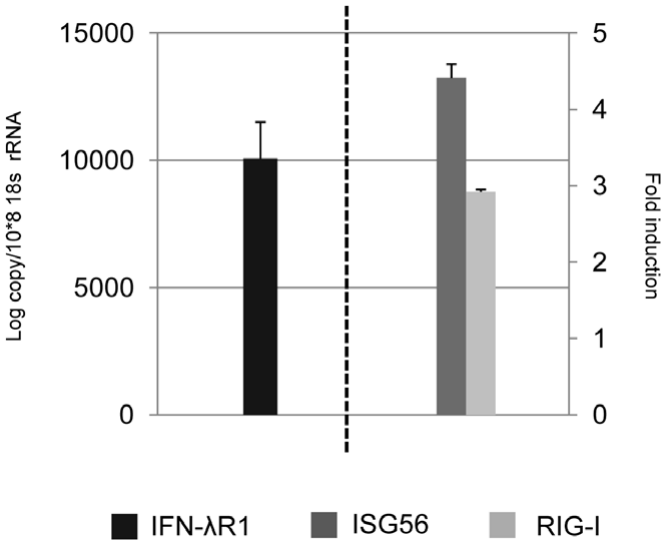
Responsiveness of *P. alecto* splenocytes to IFN-λ2 treatment and the relative expression level of IFNλR1. Cells were treated for 6 hours before they were collected for RNA extraction and qRT-PCR analysis. The left axis shows the relative expression level of IFNλR1 (mean of three experiments). The right axis shows the ISG56 and RIG-I fold induction in response to IFN-λ2 treatment (mean of three experiments). The expression level was normalized to the housekeeping gene 18s rRNA. The error bars represent standard errors.

To further investigate the cellular responsiveness to IFN-λ, we examined the response of freshly isolated bat splenocytes, a rich source of immune cells. As shown in Figure 5, the bat immune cells have much higher IFNλR1 expression compared with the tissue cell types present in the primary cell lines (Fig. 4A). Following treatment with recombinant bat IFN-λ2, the transcription of RIGI and ISG56 was upregulated over four and three fold respectively, representing a moderate ISG induction. Overall, these results demonstrate that immune cells express high levels of IFNλR1 and are responsive to IFN-λ treatment.

### IFNλR1 is a functional receptor of IFN-λ

**Figure 6:**
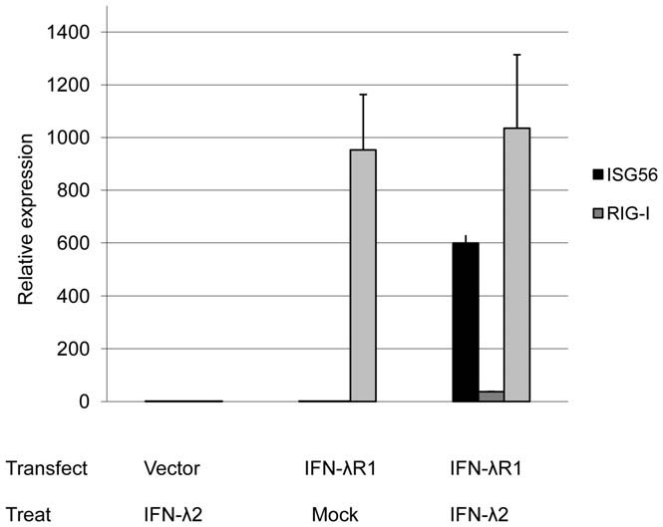
IFNλR1 is a functional receptor of IFN-λ2. Bat cloned and immortalized cell line PaKiT01 cells were transfected with IFNλR1 expression plasmid or empty vector for 24 hours, followed by IFN-λ2 treatment (or mock treatment) for another 6 hours. After which, cells were collected and total RNA was extracted for qRT-PCR analysis. IFNλR1, ISG56 and RIG-I mRNA expression were tested and indicated by relative expression (mean of two experiments). The error bars represent standard errors.

To assess whether IFNλR1 is a functional receptor for IFN-λ, we constructed a transient expression vector containing the full length ORF of IFNλR1 with a C-terminal His-tag. Successful construction was confirmed by sequencing. To demonstrate the functional activity of bat IFNλR1 in bat cells, the cloned and immortalized bat kidney cell line, PaKiT01 was transfected with IFNλR1. This cell line was chosen due to the low level of native IFNλR1 expression detected in these cells compared to other bat cell lines tested (unpublished data). Recombinant bat IFN-λ2 was used to stimulate the IFNλR1 transfected bat PaKiT01 cells for six hours and IFNλR1 and ISG induction was measured by qRT-PCR following DNaseI treatment to remove carry over plasmid DNA. As shown in Fig. 6, compared to mock transfected cells, a 1000 fold induction of IFNλR1 mRNA occurred following IFNλR1 transfection demonstrating successful transfection. Treatment of IFNλR1 transfected cells with recombinant bat IFN-λ resulted in a 600 fold induction of ISG56 and 36 fold induction of RIG-I. No ISG induction was observed in mock transfected or mock treated cells (Fig. 6).

## Discussion

Bats are rich reservoirs of emerging and re-emerging viruses [17], yet their immune response to viral infection is poorly understood. Type III IFNs are a recently discovered family of IFNs that share a number of functional characteristics with type I IFNs but signal through a different IFNR complex. Bat type III IFNs display antiviral activity similar to mammalian type I IFNs and have been demonstrated to be transcribed in virus infected bat cells despite the suppression of type I IFNs [21]. The genes encoding the bat type III IFNR complex, IFNλR1 and IL10R2 described here were identified in the publicly available whole genome sequence of the Malaysian flying fox, *P. vampyrus* and further characterized in the closely related pteropid bat, *P. alecto*. Bat IFNλR1 and IL10R2 share a similar genomic organization to their human counterparts and are highly conserved with other mammalian IFNλR genes.

In humans there are two splice variants of the IFNλR1 chain, encoding a soluble receptor and a truncated transmembrane receptor [5]. No alternative splice variants of IFNλR1 were identified in *P. alecto*, despite extensive screening of spleen and thymus RNA by RT-PCR. The two splice variants of human IFNλR1 have been speculated to negatively regulate IL-28/IL-29 signalling as a result of IFN-λ binding to the soluble IFNλR1 receptor prior to cell contact, and due to the truncated IFNλR1 acting as a dominant negative receptor chain. Although we cannot completely rule out the possibility of the presence of additional IFNλR1 splice variants in *P. alecto*, the absence of such variants with the ability to downregulate the IFN-λ response may lead to a more pronounced IFN response in bat cells.

In contrast to the ubiquitously expressed type I IFNR, there is considerable variation in type III IFNR expression between organs and even between the few species in which it has been characterised. In humans and Xenopus, the IFNλR1 chain is expressed by a range of tissues with a high level of variation, being highest in lung, heart and immune organs and lowest in the brain [1], [2], [10], [12]. In mice, IFNλR1 is expressed predominantly in stomach, small intestine and skin and is expressed at very low levels in the central nervous system and spleen [13], [16], [25]. Our results demonstrate a ubiquitous IFNλR1 expression pattern in all tissues tested in *P. alecto*, however, similar to humans and Xenopus, considerable variation exits between tissues with small intestine and spleen having relatively higher expression of IFNλR1 compared to other organs. The transcription of *P. alecto* IL10R2 was comparable to humans [25], with a ubiquitous expression among all tissues tested. Interestingly, the three hematopoietic organs tested, PBMC, spleen and lymph nodes displayed the highest IL10R2 expression likely reflecting its role as a receptor for a number of cytokines. This expression pattern correlates with the pattern of TLR7, 8 and 9 expression in *P. alecto* [20].

At the cellular level, bat IFNλR1 is capable of responding to stimulation with IFN-λ, providing evidence that it is a functional receptor. In humans and mice, IFNλR1 appears to be restricted to epithelial cells [12], [13]. To determine whether a similar situation exists in bats, we used a number of primary cell lines derived from different *P. alecto* organs and examined their IFNλR1 expression and response to IFN-λ stimulation. Our results demonstrate a clear relationship between IFNλR1 expression and response to IFN-λ stimulation. In addition, this pattern correlated with the proportion of epithelial cells in the selected bat cell lines. The restricted expression pattern of IFNλR1 in non-haematopoietic cells is similar to other species and is consistent with a role for IFN-λ in contributing to the elimination of viruses at the major portals of entry into the body before infection is established.

To investigate the role of IFN-λ in hematopoietic cells, we examined IFNλR1 expression and response to IFN-λ in freshly isolated bat splenocytes which are a rich source of immune cells. Bat spleen cells displayed a high IFNλR1 expression and responded to IFN-λ treatment with the induction of RIG-I and ISG56. However, a higher IFNλR1 expression by bat splenocytes compared with the non-hematopoietic cell lines did not correspond to a higher induction of ISGs. In mice, plasmacytoid dendritic cells are the only immune cells that express the IFNλR1 and these cells are also responsive to IFN-λ stimulation [16]. Human immune cells including B, T and NK cells express high levels of IFNλR1 mRNA, and PBMCs have membrane bound IFNλR1, however, they show little response to stimulation with IFN-λ [12]. Our results are consistent with the presence of a population of bat splenocytes that are sensitive to IFN-λ. However, in the absence of specific cell markers to characterize bat immune cells we are unable to determine which populations of cells express IFNλR1 and respond to IFN-λ. Characterization of the immune cell types capable of responding to IFN-λ during viral infection will provide further insights into the role of IFN-λ in antiviral immunity in bats.

To our knowledge, our results provide the first molecular characterization of the IFNλR system in any species of bat, demonstrating that bat IFNλR1 serves as a functional IFN-λ receptor. We previously described the upregulation of type III IFN in virus infected bat cells, despite the suppression of the type I IFN response [21]. The distribution and functional activity of IFNλR1 in both epithelial and immune cells provides additional support for an important role for IFN-λ in the antiviral immune response of bats.

## Supporting Information

Table S1 Primers used in this study.
(XLS)
